# Diagnostic and Prognostic Values of Noninvasive Predictors of Portal Hypertension in Patients with Alcoholic Cirrhosis

**DOI:** 10.1371/journal.pone.0133935

**Published:** 2015-07-21

**Authors:** Eun Ju Cho, Moon Young Kim, Jeong-Hoon Lee, Il Young Lee, Yoo Li Lim, Dae Hee Choi, Yoon Jun Kim, Jung-Hwan Yoon, Soon Koo Baik

**Affiliations:** 1 Department of Internal Medicine and Liver Research Institute, Seoul National University College of Medicine, Seoul, Korea; 2 Department of Internal Medicine, Yonsei University Wonju College of Medicine, Wonju, Korea; 3 Department of Internal Medicine, Kangwon National University Hospital, Chuncheon, Korea; IDIBAPS—Hospital Clinic de Barcelona, SPAIN

## Abstract

Portal hypertension is a direct consequence of hepatic fibrosis, and several hepatic fibrosis markers have been evaluated as a noninvasive alternative to the detection of portal hypertension and esophageal varices. In the present study, we compared the diagnostic and prognostic values of the noninvasive fibrosis markers in patients with alcoholic cirrhosis. A total of 219 consecutive alcoholic cirrhosis patients were included. Biochemical scores and liver stiffness (LS) were compared with hepatic venous pressure gradient (HVPG). For the detection of clinically significant portal hypertension (CSPH; HVPG≥10 mmHg) in compensated patients, LS and LS–spleen diameter to platelet ratio score (LSPS) showed significantly better performance with area under the curves (AUCs) of 0.85 and 0.82, respectively, than aspartate aminotransferase-to-platelet ratio index, FIB-4, Forns’ index, Lok index, (platelet count)^2^/[monocyte fraction (%) × segmented neutrophil fraction (%)], and platelet count-to-spleen diameter ratio (all *P*<0.001). However, for the detection of high-risk varices, none of the non-invasive tests showed reliable performance (AUCs of all investigated tests < 0.70). During a median follow-up period of 42.6 months, 46 patients with decompensated cirrhosis died. Lok index (hazard ratio [HR], 1.13; 95% confidence interval [CI], 1.05–1.22; *P* = 0.001) and FIB-4 (HR, 1.06; 95% CI, 1.01–1.10; *P* = 0.009) were independently associated with all-cause death in decompensated patients. Among the tested noninvasive markers, only Lok index significantly improved discrimination function of MELD score in predicting overall survival. In conclusion, LS and LSPS most accurately predict CSPH in patients with compensated alcoholic cirrhosis. In the prediction of overall survival in decompensated patients, however, Lok index is an independent prognostic factor and improves the predictive performance of MELD score.

## Introduction

Hepatic venous pressure gradient (HVPG) is one of the most important predictors of complications resulting from portal hypertension in patients with cirrhosis. Development of complications, such as esophageal varices (EVs), ascites, and hepatic encephalopathy are usually observed when the HVPG exceeds 10–12 mmHg; furthermore, a reduction below these values leads to a decrease in the risk of complications [1, 2]. Thus, HVPG measurement is important for the risk stratification of patients with cirrhosis. However, because direct measurement of HVPG is invasive and requires technical expertise, the need of noninvasive methods has become relevant.

Recently, various fibrosis markers have been evaluated as an alternative to HVPG measurement because portal hypertension depends on elevated intrahepatic vascular resistance caused by hepatic fibrosis [3]. The FibroTest, a panel of direct serum markers for hepatic fibrosis, was evaluated in this context; however, it showed only modest diagnostic performance [4]. Data on the performance of indirect serum markers composed of routine laboratory parameters are very limited. Liver stiffness (LS) measured by transient elastography showed a reasonable correlation with HVPG, particularly at HVPG values below 10 mmHg in patients with viral or alcoholic cirrhosis [5, 6] and was able to predict clinical decompensation [7]. However, further investigation of highly reproducible, less expensive markers for portal hypertension is still needed.

The aim of the present study was to evaluate which noninvasive fibrosis marker most effectively predicts portal hypertension in patients with alcoholic cirrhosis. We also examined whether combining unrelated markers such as serum markers and LS might increase diagnostic accuracy for predicting portal hypertension. In addition, the prognostic values of fibrosis markers and HVPG were compared with those of traditional risk factors for death.

## Materials and Methods

### Patients

This retrospective cohort study included patients with alcoholic cirrhosis who underwent baseline HVPG and LS measurements between January 2009 and December 2013 at Wonju Severance Christian Hospital (Wonju, Republic of Korea). Alcoholic cirrhosis was diagnosed histologically, clinically, or by typical radiological findings in patients with a history of significant alcohol consumption (at least 40 g alcohol daily for 5 years or more). Decompensated cirrhosis was defined by the presence or a previous episode of complications of cirrhosis such as hepatic encephalopathy, variceal bleeding, and ascites. Comorbidity was assessed using the Charlson Comorbidity Index [8]. Patients had to be abstinent for at least 2 month before measurements of HVPG and LS. Exclusion criteria included the presence of hepatitis B surface antigen, antibodies to hepatitis C virus, concomitant splenic or portal vein thrombosis, current use of beta-blockers, the presence of bacterial infection, other advanced complications of cirrhosis including renal and cardiopulmonary involvement, hepatocellular carcinoma, and severe comorbidity. Patients with severe ascites at time of performing transient elastography or poorly reliable liver stiffness measurements were also excluded. In total, 251 patients with alcoholic cirrhosis who underwent baseline HVPG and LS measurements were identified. Among them, 32 patients (12.7%) who could not have precise measurements of LS were excluded: 28 patients had severe ascites that might prevent the accurate assessment of LS, and four patients were severely obese. Finally, 219 patients were included in the study.

The present study conformed to the ethical guidelines of the World Medical Association Declaration of Helsinki, and was approved by the Institutional Review Board of the Yonsei University Wonju Severance Christian Hospital. Documentation of informed consent was waived by the Institutional Review Boards because of the anonymous evaluation of data.

### Measurements of HVPG, spleen diameter and LS

The HVPG was measured according to international standards, as previously described [9]. All measurements were performed at least in triplicate, and permanent tracings were obtained on a multi-channel recorder. HVPG measurement was performed by one experienced operator (YJK). The coefficient of variation of HVPG measurement was 5%. Clinically significant portal hypertension (CSPH) was defined as an HVPG ≥ 10 mmHg [1].

Within one day after or before HVPG measurement, fasting patients underwent measurement of spleen size by ultrasound, followed by LS measurement using transient elastography with a standard probe (FibroScan; Echosens). Spleen size was assessed as spleen bipolar diameter (the greatest longitudinal dimension at the level of splenic hilum) [10]. Measurement of LS was performed as previously described in detail by two experienced operators (SKB and MYK) [11]. Either fewer than 10 successful acquisitions or a success rate of < 60% was considered unreliable.

### Noninvasive markers

Laboratory examinations including complete liver function tests were performed at the day of HVPG measurement. The following noninvasive markers were evaluated according to published formulas: aspartate aminotransferase (AST)-to-platelet ratio index (APRI), Forns’ index, FIB-4, Lok index, LS–spleen diameter to platelet ratio score (LSPS), platelet count-to-spleen diameter ratio (Plt/Spl), (platelet count)^2^/[monocyte fraction (%) × segmented neutrophil fraction (%)] (P2/MS) [10, 12–17]. Because these markers, except Plt/Spl, were initially developed as hepatic fibrosis markers, cutoffs for CSPH and high-risk varices were reassessed according to receiver operating characteristic (ROC) curves analyses. The cutoff was defined as the value with the maximal sum of sensitivity and specificity [18].

### Upper endoscopic examination

The presence and size of varices were assessed according to proposed guidelines [19]. The size of the EV was classified into small, medium, and large. High-risk varices referred to small varices of red color, and medium or large varices.

### Statistical analysis

The Mann-Whitney *U* test was used to analyze differences between the different groups. The *χ* ^*2*^ test or Fisher’s exact test was used for categorical data. To assess the diagnostic performance of each noninvasive test for detection of CSPH and high-risk varices, each area under the ROC curve (AUC, equivalent to the C-statistic) was calculated. The sensitivity, specificity, positive predictive value, and negative predictive value were calculated using ROC curves.

Cox proportional hazard regression analysis was performed to evaluate independent risk factors for all-cause death. Variables with *P* < 0.1 in the univariate Cox regression analysis were subjected to multivariate analysis using forward stepwise selection. To avoid multicollinearity between the univariate variables, a correlation coefficient of < 0.7 was set. A *P* value of < 0.05 in the multivariate model was considered significant. Furthermore, the prognostic performance of each marker was assessed by C-statistic derived from time-dependent ROC curve analysis [20]. The incremental value of each noninvasive test above the model of end-stage liver disease (MELD) score was assessed by the difference in C-statistic [21], the integrated discrimination improvement (IDI), and the continuous net reclassification improvement (NRI) which is independent of pre-specified risk cutoffs and maximizes statistical power [22]. The analyses were performed using PASW 18.0K (SPSS, Chicago, IL) and the R language environment, version 3.0.2 (http://www.r-project.org).

## Results

### Baseline patient characteristics and noninvasive markers

Main clinical characteristics of the 219 patients are presented in Table 1. A total of 88 patients (40.2%) had compensated cirrhosis and 131 (59.8%) had decompensated cirrhosis. Among patients with compensated cirrhosis, 44 (50.0%) had CSPH, and 10 (11.4%) had high-risk varices. Forty of 88 compensated patients and 117 of 131 decompensated patients received prophylactic non-selective beta-blockers and/or endoscopic variceal ligation (EVL). S1 Table shows detailed characteristics and treatment outcomes according to the use of prophylactic beta-blockers and/or EVL. As expected, patients receiving prophylactic treatment exhibited higher HVPG and had more advanced liver disease than control, as indicated by increased prevalence of high-risk varices, higher Child-Pugh and MELD scores. There was no patient who was treated for alcoholic hepatitis at the time of HVPG and LS measurements. Overall, 39 patients (9 in the compensated group, 30 in the decompensated group) developed acute-on-chronic liver failure [23] during the follow-up period, and 13 cases of them (1 in the compensated group, 12 in the decompensated group) developed due to active alcohol consumption. Among them, five patients received steroids or pentoxifylline; however, none of them responded to treatment.

**Table 1:** Main clinical data of patients according to the compensated or decompensated stage of cirrhosis.

	Compensated patients (n = 88)	Decompensated patients (n = 131)	*P*
Age, years	52 (46–58)	50 (44–56)	0.12
Male, n (%)	78 (88.6)	123 (93.9)	0.21
Abstinence during follow-up, n (%)	60 (68.2)	76 (58.0)	0.16
Number of previous decompensation events, n (%)			
None	88 (100.0)	18 (13.7)	
1	-	100 (76.3)	
2	-	8 (6.1)	
≥3	-	5 (3.9)	
Types of previous decompensation events, n (%)			
Ascites	-	32 (24.5)	
Hepatic encephalopathy	-	9 (6.9)	
Variceal bleeding	-	54 (41.2)	
Multiple events	-	17 (13.0)	
WBC, mm^-3^	4940 (4030–6827)	4940 (3430–6130)	0.18
Platelet count, 10^9^/L	154 (96–233)	123 (80–178)	0.01
AST, IU/L	51 (32–73)	62 (42–84)	0.03
ALT, IU/L	29 (19–49)	23 (15–41)	0.04
GGT, U/L	290 (117–433)	188 (91–433)	0.14
Albumin, g/dL	3.6 (3.3–3.9)	3.2 (3.0–3.6)	< 0.001
Bilirubin, mg/dL	0.8 (0.5–1.4)	1.3 (0.7–2.3)	< 0.001
Prothrombin time, INR	1.0 (0.9–1.1)	1.1 (1.0–1.3)	< 0.001
Creatinine, mg/dL	0.6 (0.5–0.8)	0.7 (0.6–0.8)	0.5
Spleen diameter, cm	10.7 (9.7–11.8)	12.0 (10.7–13.8)	< 0.001
Child-Pugh class, n (%)			< 0.001
A	74 (84.1)	56 (42.7)	
B	14 (15.9)	65 (49.6)	
C	0	10 (7.6)	
MELD score	8 (7–10)	10 (8–13)	< 0.001
HVPG, mmHg	9 (6–13)	15 (11–18)	< 0.001
Esophageal varices, n (%)			< 0.001
None	60 (68.2)	36 (27.5)	
Small	21 (23.9)	41 (31.3)	
Medium	5 (5.7)	49 (37.4)	
Large	2 (2.3)	5 (3.8)	
Gastric varices, n (%)			0.01
None	74 (84.1)	87 (66.4)	
Small	10 (11.4)	27 (20.6)	
Medium	4 (4.5)	14 (10.7)	
Large	0	3 (2.3)	
High-risk varices, n (%)	10 (11.4)	63 (48.1)	< 0.001
Charlson Comorbidity Index	1 (1–2)	3 (2–3)	< 0.001
Prophylactic treatment, n (%)			< 0.001
Beta-blockers	39 (44.3)	85 (64.9)	
Band ligation	1 (1.1)	2 (1.5)	
Both beta-blockers and band ligation	0	30 (22.9)	

Data are median (interquartile range) or number (%), unless otherwise indicated.

AST, aspartate aminotransferase; ALT, alanine aminotransferase; GGT, gamma-glutamyltransferase; HVPG, hepatic venous pressure gradient; INR, international normalized ratio; MELD, model of end-stage liver disease.

The median values obtained with different noninvasive markers according to the clinical course of cirrhosis, presence of CSPH and high-risk varices are presented in S2 Table. Lok index, LS, and LSPS were significantly different between patients with compensated and decompensated cirrhosis (all *P*<0.001). Statistically significant differences were observed for all tested noninvasive markers according to the presence of both CSPH and high-risk varices.

### Performances of noninvasive markers for detection of CSPH and high-risk varices

**Fig 1:**
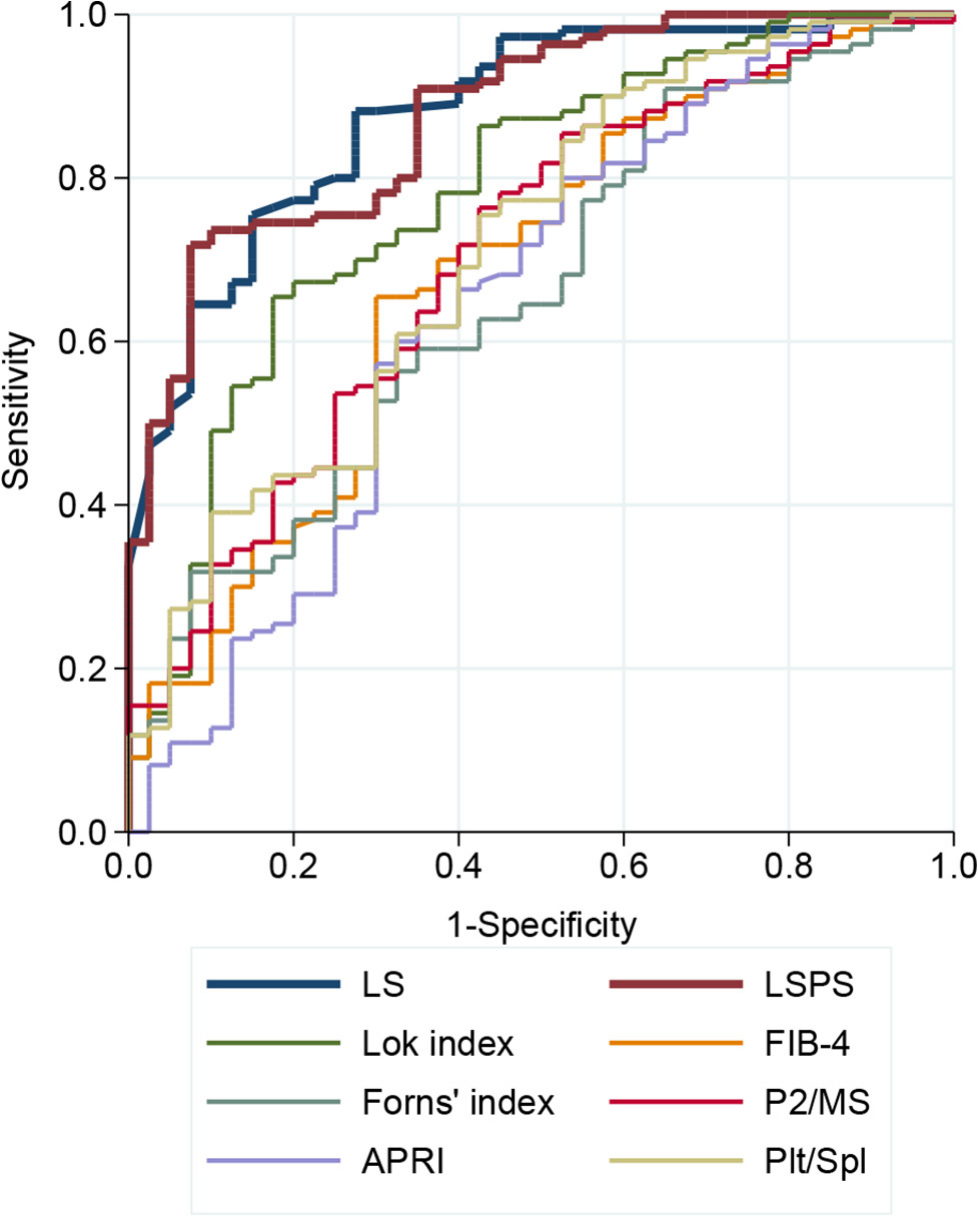
ROC curves of noninvaisve fibrosis tests for the detection of clinically significant portal hypertension in compensated patients. The AUCs of LS (0.85; 95% CI, 0.74–0.95) and LSPS (0.82; 95% CI, 0.71–0.93) were significantly higher than those of other noninvasive markers (all *P*<0.001).

The diagnostic performances of noninvasive markers for detection of CSPH and high-risk varices are presented in S3 Table. LS and LSPS showed the best performances for detection of CSPH in compensated patients as indicated by AUCs of 0.85 (95% confidence interval [CI], 0.74–0.95) and 0.82 (95% CI, 0.71–0.93), respectively (Fig 1). Overall, the AUCs of LS and LSPS were significantly higher with respect to those of the APRI, FIB-4, Forns’ index, Lok index, P2/MS, and Plt/Spl (all *P*<0.001). There was no significant difference between the AUCs of LS and LSPS (*P* = 0.76). None of the tested serum markers add additional diagnostic value in combination with LS or LSPS.

However, for the detection of high-risk varices, none of the noninvasive tests showed reliable performance (AUCs of all investigated tests < 0.70). Only invasive HVPG measurement showed moderate performance (AUC, 0.79; 95% CI, 0.67–0.90), which was significantly greater than those of the APRI (*P* = 0.001), FIB-4 (*P* = 0.008), Forns’ index (*P* = 0.01), P2/MS (*P* = 0.02), and LS (*P* = 0.02). Although the AUCs of Plt/Spl, Lok index, and LSPS were not statistically different from that of HVPG, they did not show sufficient performances (all AUCs ≦ 0.65). Combination of any of these tests did not improve the diagnostic value for detection of high-risk varices.

When considering all patients, LS and LSPS showed the best performance for detection of CSPH (AUC = 0.88 and 0.87, respectively). However, none of the noninvasive tests showed reliable performance in the identifying high-risk varices (all AUCs < 0.70).

### Performances of noninvasive markers for predicting risk of death

Among 88 patients with compensated cirrhosis, 12 (13.6%) patients experienced variceal bleeding and 22 (25.0%) developed clinical decompensation during the follow-up period (median, 42.6 months; interquartile range, 28.2–58.0 months). Bleeding episodes did not differ according the use of prophylactic treatment (*P* = 0.11), and prophylactic treatment was not significantly associated with the risk of decompensation (*P* = 0.10). With regard to overall mortality, 64 patients (18 in the compensated group; 46 in the decompensated group) died during follow-up. Twenty-three deaths were attributable to liver disease and three were due to non-liver-related causes. The cause of death could not be assessed in 38 cases due to follow-up loss. Because the number of liver-related complications such as decompensation and liver-related death was too small to construct a robust model, we analyzed only all-cause death of decompensated patients that are those at the highest risk of liver-related death.

The MELD score as a continuous variable, albumin, HVPG, and noninvasive markers such as the FIB-4, Lok index, LSPS, LS and Plt/Spl were significantly associated with overall survival (OS) of decompensated patients in the univariate analyses (Table 2). Alcohol consumption and prophylactic treatment did not have major impact on outcome (*P* = 0.07 and 0.12, respectively). The correlation coefficients between LSPS and LS, LSPS and Plt/Spl were 0.75 and 0.76, respectively (both *P*<0.001), whereas the correlation coefficient between LS and Plt/Spl was –0.18 (*P*<0.01). Considering the multicollinearity between LS, Plt/Spl and LSPS, only LSPS was included in the final model. Significant prognostic factors for OS were Lok index (hazard ratio [HR], 1.13; 95% CI, 1.05–1.22; *P* = 0.001) and FIB-4 (HR, 1.06; 95% CI, 1.01–1.10; *P* = 0.009), without independent prognostic values for LSPS and HVPG. We further evaluated whether a model including noninvasive fibrosis markers or HVPG, in combination with the well-known prognostic factor, the MELD score, may provide additional value in predicting 3-year mortality (Table 3). Among the tests, only Lok index significantly improved the predictive ability of the MELD score in both discrimination (difference in C-statistic, 0.049; 95% CI, 0.001–0.096) and classification (IDI, 0.064; 95% CI, 0.021–0.126; *P*<0.001; NRI, 0.258; 95% CI, 0.022–0.388; *P* = 0.03). Combination of other fibrosis markers or HVPG with the MELD score did not significantly improve the prognostic value of the MELD score alone.

**Table 2:** Univariate and multivariate analyses of factors associated with overall survival.

	Univariate analysis		Multivariate analysis	
	HR (95% CI)	*P*	HR (95% CI)	*P*
MELD	1.083 (1.009–1.163)	0.03	-	0.19
Albumin	0.434 (0.244–0.770)	0.004	-	0.24
HVPG	1.071 (1.013–1.132)	0.02	-	0.47
FIB-4	1.082 (1.042–1.123)	< 0.001	1.059 (1.014–1.106)	0.009
Lok index	1.167 (1.093–1.247)	< 0.001	1.131 (1.051–1.217)	0.001
LSPS	1.060 (1.010–1.113)	0.02	-	0.73
Plt/Spl	0.945 (0.897–0.996)	0.04		
LS	1.011 (0.997–1.024)	0.13		

CI, confidence interval; HR, hazard ratio; HVPG, hepatic venous pressure gradient; LS, liver stiffness; LSPS, liver stiffness–spleen diameter to platelet ratio score; MELD, model of end-stage liver disease; Plt/Spl, platelet count-to-spleen diameter ratio; P2/MS, (platelet count)2/[monocyte fraction (%) × segmented neutrophil fraction (%)].

**Table 3:** Accuracy of each test in the prediction of 3-year mortality when added to MELD score.

Variables	C-statistic	Difference in C (95% CI)	IDI	*P*	NRI	*P*
MELD score	0.630					
MELD score + HVPG	0.654	0.025 (-0018–0.068)	0.039 (0.004–0.103)	0.024	0.093 (-0.052–0.281)	0.216
MELD score + Plt/Spl	0.665	0.035 (-0.012–0.082)	0.024 (-0.004–0.074)	0.110	0.199 (-0.031–0.350)	0.082
MELD score + P2/MS	0.679	0.050 (-0.009–0.108)	0.019 (-0.011–0.082)	0.302	0.103 (-0.123–0.296)	0.370
MELD score + FIB4	0.661	0.031 (-0.018–0.080)	0.029 (-0.003–0.095)	0.100	0.156 (-0.026–0.327)	0.082
MELD score + Forns' index	0.657	0.027 (-0.028–0.082)	0.007 (-0.012–0.061)	0.631	0.056 (-0.166–0.256)	0.517
MELD score + Lok index	0.678	0.049 (0.001–0.096)	0.064 (0.021–0.126)	< 0.001	0.258 (0.022–0.388)	0.030
MELD score + LS	0.636	0.006 (-0.028–0.039)	0.018 (-0.003–0.080)	0.148	0.119 (-0.118–0.275)	0.230
MELD score + LSPS	0.648	0.019 (-0.014–0.052)	0.015 (-0.006–0.059)	0.202	0.154 (-0.076–0.319)	0.146

CI, confidence interval; HVPG, hepatic venous pressure gradient; IDI, integrated discrimination improvement; LS, liver stiffness; LSPS, liver stiffness–spleen diameter to platelet ratio score; MELD, model of end-stage liver disease; NRI, net reclassification improvement; Plt/Spl, platelet count-to-spleen diameter ratio; P2/MS, (platelet count)2/[monocyte fraction (%) × segmented neutrophil fraction (%)].

## Discussion

The present study showed that, among the easily available noninvasive fibrosis tests, LS and LSPS most accurately predicted CSPH in patients with compensated alcoholic cirrhosis; however, their performance for the diagnosis of high-risk varices did not differ from those of other tests. Combination of any of these tests did not improve the diagnostic value for detection of CSPH or high-risk varices. In regard to the prognostic values for predicting death in decompensated patients, Lok index was independently associated with OS, and significantly improved accuracy of the traditional prognostic factor, MELD score.

Indirect fibrosis markers validated in staging hepatic fibrosis have the benefit of availability and noninvasiveness; therefore, they are appropriate for screening. However, only a few markers have been evaluated in regard to the detection of portal hypertension. FibroTest is the only patent biochemical tests evaluated for diagnosis of severe portal hypertension; a study showed an AUC of 0.79 [4], which has not been reconfirmed. According to a recent study [24], the Lok index showed a reliable performance for detection of CSPH in compensated cirrhosis (AUC = 0.83); which was similar to the result of our study. However, all tested serum fibrosis markers were inferior to LS and LSPS as a single test, and did not add diagnostic value in combination with them in the present study.

A good correlation between LS and HVPG, especially with HVPG values below 10 mm Hg, has been reported [5, 6], and LS appears to be useful in detecting the presence of CSPH. Consistent with previous reports, LS outperformed other serum markers in identifying CSPH in our study. In addition, LSPS, which is a score developed for diagnosing cirrhosis and high-risk esophageal varices in patients with hepatitis B virus-related chronic liver disease [10, 25], recently exhibited a good performance for detecting CSPH [26]; and this finding was also confirmed in our study. Thus, if LS and LSPS can be successfully measured, they might be the most useful tests for assessing CSPH among the readily available noninvasive fibrosis markers. However, the approximately 20% technical failure rate of LS should be taken into consideration [27].

In spite of its usefulness for the detection of CSPH, LS did not show reliable performance in the prediction of high-risk varices. This is not surprising, because LS does not significantly correlate with portal hypertension beyond a certain degree of HVPG (10–12 mmHg) [5], above which varices may start to develop [27]. In addition, portal hypertension depends on not only static fibrosis components, but also to hemodynamic components, which correlate with splanchnic and portal venous blood flow; thus, a fibrosis marker alone may be insufficient for detecting a portal hypertension-induced complication. Previously, LSPS showed a good performance for discrimination of varices or high-risk varices in compensated cirrhosis [10, 26]. However, in the present study, LSPS failed to perform well. This might depend on the differences in the etiologies of underlying chronic liver disease (mainly viral cirrhosis in the previous reports vs. alcoholic cirrhosis in the present study), and possible effects of alcohol drinking on the platelet count or LS measurement [28, 29], although the performance of LSPS was not significantly different in abstinence patients.

Regarding the prognostic value, HVPG and LS has been reported as predictors of hepatic decompensation and death in patients with chronic liver disease [1, 30]. However, HVPG and LS were not independently associated with OS in the present study. Furthermore, combination of HVPG or LS with the MELD score did not significantly improve the prognostic value of the MELD score alone. This is probably because, the grade of hepatic dysfunction and complications induced by portal hypertension in themselves, rather than the degree of portal hypertension or hepatic fibrosis, may provide more important information to predict survival in patients with alcoholic cirrhosis.

It is interesting that Lok index, a fibrosis marker based on AST/ALT ratio, PT-INR, and platelet count, was predictive of clinical outcome in patients with alcoholic cirrhosis. Recent studies reported that Lok index showed a reliable performance for the diagnosis of high-risk varices when combined with the Forns’ index in decompensated cirrhosis [31]; and was independently related to the degree of portal hypertension [24]. However, there has been no study regarding its prognostic value. In the present study, Lok index was independently associated with OS, and improved the predictive ability of the MELD score. The AST/ALT ratio included in Lok index is associated with advanced fibrosis in alcoholic liver disease [32]; PT-INR is related to hepatic insufficiency; and platelet count reflects splenomegaly and portal hypertension. Because Lok index uses continuous variables, subtle changes in variables related to hepatic insufficiency and advanced disease may lead to our finding that Lok index was an independent predictor of survival. Whether this result might be applied to cirrhotic patients with different etiologies remains to be determined.

The present study has several limitations. First, the presence of alcoholic steatohepatitis, a potential confounding factor affecting LS [27], could not be assessed due to lack of histological data. However, LS and HVPG measurements were performed in patients who had been abstinent for at least 2 month to avoid the influence of active inflammation, and the diagnostic and prognostic values of LS and LSPS did not change significantly according to the abstinence state. Second, associations between baseline noninvasive markers and relevant liver-related outcomes could not be assessed because of the small number of events, and inaccessibility to the cause of death in several cases. Third, the relationship between longitudinal changes in noninvasive markers and outcomes could not be examined due to lack of serial measurements of variables. Further prospective cohort studies are warranted to solve this question.

In conclusion, among the noninvasive fibrosis tests, LS and LSPS most accurately predict CSPH in patients with alcoholic cirrhosis; however, they did not show independent prognostic values. In contrast, Lok index was independently associated with survival and improved the predictive performance of the MELD score.

## Supporting Information

S1 Table Baseline characteristics and treatment outcomes according to the use of prophylactic beta-blockers and/or endoscopic variceal ligation. (DOCX)

S2 Table Values of noninvasive tests according to the compensated state, degree of portal hypertension, and presence of high-risk varices. (DOCX)

S3 Table Diagnostic performance of non-invasive tests for the detection of clinically significant portal hypertension and high-risk varices in patients with compensated cirrhosis. (DOCX)
