# Supplementary material for: Diagnostic and Prognostic Values of Noninvasive Predictors of Portal Hypertension in Patients with Alcoholic Cirrhosis
Source: PLoS One. 2015 Jul 21;10(7):e0133935. doi: 10.1371/journal.pone.0133935 (PMC4511411; doi:10.1371/journal.pone.0133935)
Supplement: S1 Table — (DOCX) [file pone.0133935.s001.docx]

S1 Table. Baseline characteristics and treatment outcomes according to the use of prophylactic beta-blockers and/or endoscopic variceal ligation

|  | Compensated patients | | | Decompensated patients | | |
| --- | --- | --- | --- | --- | --- | --- |
| Characteristic | No prophylaxis group (n=48) | Prophylaxis group (n=40) | *P* | No prophylaxis group (n=14) | Prophylaxis group (n=117) | *P* |
| Admission |  |  |  |  |  |  |
| Age, years | 53 (44-59) | 52 (47-56) | 0.74 | 49 (39-55) | 50 (44-56) | 0.55 |
| Male, n (%) | 42 (87.5) | 36 (90.0) | 0.75 | 14 (100.0) | 109 (93.2) | 0.6 |
| Abstinence during follow-up, n (%) | 31 (64.6) | 29 (72.5) | 0.50 | 11 (78.6) | 65 (55.6) | 0.15 |
| Number of previous decompensation events, n (%) |  |  |  |  |  | 0.54 |
| None | 48 (100.0) | 40 (100.0) |  | 2 (14.3) | 16 (13.7) |  |
| 1 |  |  |  | 12 (85.7) | 88 (75.2) |  |
| ≥2 |  |  |  | 0 | 13 (11.1) |  |
| Types of previous decompensation events, n (%) |  |  |  |  |  | 0.20 |
| Ascites | - | - |  | 5 (35.7) | 27 (23.1) |  |
| Hepatic encephalopathy | - | - |  | 2 (14.3) | 7 (6.0) |  |
| Variceal bleeding | - | - |  | 2 (14.3) | 52 (44.4) |  |
| Multiple events | - | - |  | 3 (21.4) | 14 (12.0) |  |
| WBC, mm^-3^ | 4895 (4040-6050) | 5080 (3977-8087) | 0.31 | 5590 (4250-7430) | 4650 (3365-6085) | 0.18 |
| Platelet count, 10^9^/L | 162 (96-253) | 134 (92-206) | 0.24 | 174 (88-218) | 119 (79-162) | 0.07 |
| AST, IU/L | 50 (30-71) | 51 (36-82) | 0.40 | 63 (40-109) | 62 (43-83) | 0.85 |
| ALT, IU/L | 33 (20-51) | 26 (17-43) | 0.13 | 21 (18-61) | 23 (15-40) | 0.63 |
| GGT, U/L | 262 (129-398) | 304 (111-521) | 0.58 | 347 (83-836) | 188 (91-362) | 0.49 |
| Albumin, g/dL | 3.7 (3.4-4.0) | 3.4 (3.1-3.8) | 0.01 | 3.3 (2.8-3.9) | 3.2 (3.0-3.5) | 0.84 |
| Bilirubin, mg/dL | 0.7 (0.4-1.1) | 1.0 (0.6-2.0) | 0.05 | 1.7 (0.6-5.0) | 1.2 (0.7-2.2) | 0.36 |
| Prothrombin time, INR | 1.0 (1.0-1.1) | 1.1 (1.0-1.3) | 0.001 | 1.1 (0.9-1.5) | 1.1 (1.0-1.3) | 0.41 |
| Creatinine, mg/dL | 0.7 (0.5-0.8) | 0.6 (0.5-0.8) | 0.75 | 0.7 (0.6-0.9) | 0.7 (0.6-0.8) | 0.06 |
| Spleen diameter, cm | 10.7 (9.1-12.0) | 10.9 (10.0-11.8) | 0.47 | 11.7 (10.3-14.0) | 12.0 (10.9-13.8) | 0.64 |
| Child-Pugh class, n (%) |  |  | 0.02 |  |  | 0.45 |
| A | 45 (93.8) | 29 (72.5) |  | 7 (50.0) | 49 (41.9) |  |
| B | 3 (6.3) | 11 (27.5) |  | 5 (35.7) | 60 (51.3) |  |
| C | 0 | 0 |  | 2 (14.3) | 8 (6.8) |  |
| MELD score | 7 (6-8) | 9 (7-10) | 0.002 | 9 (6-18) | 10 (8-13) | 0.61 |
| Child-Pugh score | 5 (5-6) | 6 (5-7) | 0.001 | 7 (5-9) | 7 (6-8) | 0.86 |
| HVPG, mmHg | 7 (6-9) | 13 (11-16) | < 0.001 | 9 (5-13) | 15 (12-18) | < 0.001 |
| Esophageal varices, n (%) |  |  | 0.08 |  |  | 0.04 |
| None | 38 (79.2) | 22 (55.0) |  | 8 (57.1) | 28 (23.9) |  |
| Small | 8 (16.7) | 13 (32.5) |  | 3 (21.4) | 38 (32.5) |  |
| Medium | 1 (2.1) | 4 (10.0) |  | 2 (14.3) | 47 (40.2) |  |
| Large | 1 (2.1) | 1 (2.5) |  | 1 (7.1) | 4 (3.4) |  |
| Gastric varices, n (%) |  |  | 0.10 |  |  | 0.01 |
| None | 44 (91.7) | 30 (75.0) |  | 14 (100.0) | 73 (62.4) |  |
| Small | 3 (6.3) | 7 (17.5) |  | 0 | 27 (23.1) |  |
| Medium | 1 (2.1) | 3 (7.5) |  | 0 | 14 (12.0) |  |
| Large | 0 | 0 |  | 0 | 3 (2.6) |  |
| High-risk varices, n (%) | 2 (4.2) | 8 (20.0) | 0.02 | 3 (21.4) | 60 (51.3) | 0.04 |
| Charlson Comorbidity Index | 2 (1-2) | 1 (1-2) | 0.05 | 3 (1-3) | 3 (2-3) | 1.00 |
| Outcomes |  |  |  |  |  |  |
| Acute decompensation, n (%) |  |  |  |  |  |  |
| Variceal bleeding | 4 (8.3) | 8 (20.0) | 0.11 | 1 (7.1) | 34 (29.1) | 0.11 |
| Ascites | 3 (6.3) | 7 (17.5) | 0.18 | 0 | 13 (11.1) | 0.36 |
| Hepatic encephalopathy | 3 (6.3) | 3 (7.5) | 1.00 | 3 (21.4) | 15 (12.8) | 0.41 |
| Spontaneous bacterial peritonitis | 0 | 1 (2.5) | 0.46 | 1 (7.1) | 0 | 0.11 |
| Hepatorenal syndrome | 0 | 3 (7.5) | 0.09 | 0 | 4 (3.4) | 1.00 |
| Overall mortality, n (%) | 6 (12.5) | 12 (30.0) | 0.04 | 2 (14.3) | 44 (37.6) | 0.14 |

Unless otherwise indicated, data are medians, and data in parentheses are interquartile ranges.
