# Supplementary material for: Diagnostic and Prognostic Values of Noninvasive Predictors of Portal Hypertension in Patients with Alcoholic Cirrhosis
Source: PLoS One. 2015 Jul 21;10(7):e0133935. doi: 10.1371/journal.pone.0133935 (PMC4511411; doi:10.1371/journal.pone.0133935)
Supplement: S2 Table — (DOCX) [file pone.0133935.s002.docx]

S2 Table. Values of noninvasive tests according to the compensated state, degree of portal hypertension, and presence of high-risk varices

|  | Compensated patients (n=88) | Decompensated patients (n=131) | *P* | HVPG <10 mmHg (n=58) | HVPG ≥10 mmHg (n=161) | *P* | Patients without high-risk varices (n=146) | Patients with high-risk varices (n=73) | *P* |
| --- | --- | --- | --- | --- | --- | --- | --- | --- | --- |
| APRI | 0.8 (0.5-1.6) | 1.3 (0.9-2.2) | < 0.001 | 0.8 (0.4-1.3) | 1.3 (0.8-2.1) | < 0.001 | 1.0 (0.6-1.9) | 1.3 (0.9-2.0) | 0.05 |
| FIB-4 | 3.1 (2.0-6.0) | 4.9 (3.5-8.5) | < 0.001 | 2.7 (1.8-4.8) | 4.9 (3.0-7.7) | < 0.001 | 4.2 (2.1-6.7) | 4.9 (3.3-7.9) | 0.02 |
| Forns' index | 8.2 (5.7-9.6) | 8.8 (7.5-10.5) | 0.02 | 8.3 (5.4-9.4) | 8.8 (7.5-10.5) | 0.01 | 8.3 (6.8-9.9) | 9.1 (7.9-11.2) | 0.02 |
| Lok index | 0.9 (-0.4-2.1) | 2.5 (1.2-4.2) | < 0.001 | 0.3 (-0.7-1.4) | 2.4 (1.0-3.9) | < 0.001 | 1.5 (0.2-3.2) | 2.5 (1.0-3.8) | 0.004 |
| P2/MS | 50.1 (22.3-120.9) | 25.3 (10.8-60.8) | 0.002 | 65.2 (22.9-196.7) | 26.4 (11.9-60.2) | 0.001 | 39.3 (19.2-98.9) | 20.2 (9.0-48.6) | 0.007 |
| Plt/Spl | 13.4 (8.2-21.9) | 9.7 (5.9-14.7) | < 0.001 | 16.6 (9.7-24.8) | 9.9 (6.1-14.7) | < 0.001 | 12.6 (7.8-21.3) | 9.5 (5.4-12.4) | < 0.001 |
| LS (kPa) | 22.1 (12.0-34.4) | 36.3 (26.4-66.4) | < 0.001 | 14.3 (10.3-24.2) | 36.1 (26.3-64.2) | < 0.001 | 27.9 (16.9-48.0) | 35.3 (24.5-66.4) | 0.004 |
| LSPS | 1.4 (0.7-3.9) | 4.0 (2.5-6.4) | < 0.001 | 1.0 (0.4-2.3) | 4.1 (2.3-6.9) | < 0.001 | 2.4 (1.0-5.3) | 4.4 (2.8-7.5) | < 0.001 |

Data are medians, and data in parentheses are interquartile ranges.

APRI, aspartate aminotransferase-to-platelet ratio index; EVs, esophageal varices; HVPG, hepatic venous pressure gradient; LS, liver stiffness; LSPS, liver stiffness –spleen diameter to platelet ratio score; Plt/Spl, platelet count-to-spleen diameter ratio; P2/MS, (platelet count)2/[monocyte fraction (%) × segmented neutrophil fraction (%)].
