# Supplementary material for: Diagnostic and Prognostic Values of Noninvasive Predictors of Portal Hypertension in Patients with Alcoholic Cirrhosis
Source: PLoS One. 2015 Jul 21;10(7):e0133935. doi: 10.1371/journal.pone.0133935 (PMC4511411; doi:10.1371/journal.pone.0133935)
Supplement: S3 Table — (DOCX) [file pone.0133935.s003.docx]

S3 Table. Diagnostic performance of non-invasive tests for the detection of clinically significant portal hypertension and high-risk varices in patients with compensated cirrhosis

|  | Noninvasive tests | Cutoff | Sensitivity (%) | Specificity (%) | PPV (%) | NPV (%) | Positive likelihood ratio | Negative likelihood ratio | AUC (95% CI) |
| --- | --- | --- | --- | --- | --- | --- | --- | --- | --- |
| CSPH | APRI | 1.0 | 55.8 | 67.5 | 64.9 | 58.7 | 1.8 | 0.6 | 0.64 (0.50-0.79) |
|  | FIB-4 | 4.1 | 53.5 | 72.5 | 67.7 | 59.2 | 2.4 | 0.6 | 0.65 (0.50-0.80) |
|  | Forns' index | 8.9 | 44.8 | 69.2 | 61.9 | 52.9 | 1.6 | 0.7 | 0.64 (0.49-0.79) |
|  | Lok index | 0.8 | 72.1 | 70.0 | 72.1 | 70 | 2.5 | 0.4 | 0.76 (0.64-0.89) |
|  | P2/MS | 60.2 | 69.0 | 57.7 | 64.5 | 62.5 | 1.7 | 0.5 | 0.67 (0.53-0.82) |
|  | Plt/Spl | 10.2 | 53.5 | 77.5 | 71.9 | 60.8 | 2.5 | 0.6 | 0.68 (0.53-0.82) |
|  | LS (kPa) | 21.8 | 72.1 | 70.0 | 72.1 | 70.0 | 2.4 | 0.4 | 0.85 (0.74-0.95) |
|  | LSPS | 1.7 | 62.8 | 72.5 | 71.1 | 64.4 | 2.4 | 0.5 | 0.82 (0.71-0.93) |
| High-risk varices | APRI | 1.2 | 30.0 | 68.0 | 10.7 | 88.3 | 1.2 | 0.9 | 0.42 (0.24-0.59) |
|  | FIB-4 | 2.6 | 70.0 | 42.3 | 13.5 | 91.7 | 1.9 | 0.5 | 0.56 (0.40-0.73) |
|  | Forns' index | 9.1 | 25.0 | 68.5 | 5.6 | 92.5 | 1.6 | 0.7 | 0.52 (0.26-0.78) |
|  | Lok index | 1.5 | 60.0 | 64.1 | 17.7 | 92.6 | 1.7 | 0.6 | 0.65 (0.48-0.83) |
|  | P2/MS | 69.4 | 75.0 | 37.0 | 8.1 | 95.2 | 1.2 | 0.7 | 0.47 (0.23-0.71) |
|  | Plt/Spl | 14.9 | 70.0 | 48.7 | 14.9 | 92.7 | 1.6 | 0.4 | 0.65 (0.49-0.80 |
|  | LS (kPa) | 24.0 | 50.0 | 56.4 | 12.8 | 90.0 | 1.1 | 0.9 | 0.55 (0.34-0.76) |
|  | LSPS | 7.4 | 40.0 | 94.9 | 50.0 | 92.5 | 7.8 | 0.6 | 0.63 (0.43-0.82) |

APRI, aspartate aminotransferase-to-platelet ratio index; AUC, area under the receiver operating characteristic curve; CI, confidence interval; CSPH, clinically significant portal hypertension; EVs, esophageal varices; HVPG, hepatic venous pressure gradient; LS, liver stiffness; LSPS, liver stiffness–spleen diameter to platelet ratio score; NPV, negative predictive value; PPV, positive predictive value; Plt/Spl, platelet count-to-spleen diameter ratio; P2/MS, (platelet count)2/[monocyte fraction (%) × segmented neutrophil fraction (%)].
